# Melatonin inhibits HCC progression through regulating the alternative splicing of NEMO

**DOI:** 10.3389/fphar.2022.1007006

**Published:** 2022-09-26

**Authors:** Lu Bai, Siwen Sun, Wenmei Su, Chaoqun Chen, Yuesheng Lv, Jinrui Zhang, Jinyao Zhao, Man Li, Yangfan Qi, Wenjing Zhang, Yang Wang

**Affiliations:** ^1^ Institute of Cancer Stem Cell, Dalian Medical University, Dalian, China; ^2^ Department of Oncology, The Second Affiliated Hospital of Dalian Medical University, Dalian, China; ^3^ Department of Pulmonary Oncology, Affiliated Hospital of Guangdong Medical University, Zhanjiang, China

**Keywords:** melatonin, HCC, NEMO, alternative splicing, MT1

## Abstract

Hepatocellular carcinoma (HCC) is one of the most common primary cancers with limited therapeutic options. Melatonin, a neuroendocrine hormone produced primarily by the pineal gland, demonstrates an anti-cancer effect on a myriad of cancers including HCC. However, whether melatonin could suppress tumor growth through regulating RNA alternative splicing remains largely unknown. Here we demonstrated that melatonin could inhibit the growth of HCC. Mechanistically, melatonin induced transcriptional alterations of genes, which are involved in DNA replication, DNA metabolic process, DNA repair, response to wounding, steroid metabolic process, and extracellular matrix functions. Importantly, melatonin controlled numerous cancer-related RNA alternative splicing events, regulating mitotic cell cycle, microtubule-based process, kinase activity, DNA metabolic process, GTPase regulator activity functions. The regulatory effect of melatonin on alternative splicing is partially mediated by melatonin receptor MT1. Specifically, melatonin regulates the splicing of *IKBKG* (NEMO), an essential modulator of NF-κB. In brief, melatonin increased the production of the long isoform of NEMO-L with exon 5 inclusion, thereby inhibiting the growth of HepG2 cells. Collectively, our study provides a novel mechanism of melatonin in regulating RNA alternative splicing, and offers a new perspective for melatonin in the inhibition of cancer progression.

## Introduction

Liver cancer is the sixth most common cancer, and the third leading cause of cancer death all over the world (Sung et al., 2021). HCC accounts for about 80% of liver cancers, which has poor prognosis and is the major cause of liver cancer-related mortality (Yang et al., 2019). The current treatment strategies for HCC includes surgical resection, chemotherapy, hormonal therapy, liver transplantation and percutaneous local ablation. Due to the absence of specific symptoms in early stages and the lack of diagnostic markers, more than 70% of patients with HCC are often diagnosed in an advanced stage (Llovet et al., 2016; Montella et al., 2016; Liu and Qin, 2019). The multiple-target tyrosine kinase inhibitor sorafenib is one of the most effective drugs for the advanced HCC (Yang et al., 2019). However, only about 30% of patients can benefit from sorafenib treatment, which might lead to drug resistance and side effects as well. HCC is a heterogeneous tumor, containing alterations of multiple signaling pathway. The complex patho-physiology of HCC urgently drives the discovery of new treatment or combination therapy (Huang et al., 2020; Tang et al., 2020).

Melatonin (N-acetyl-5-methoxytryptamine), a neuroendocrine hormone produced primarily by the pineal gland, has various functions, including regulating circadian rhythm and antioxidant. Melatonin can activate melatonin receptor MT1, MT2, MT3, and ROZ/ROR to function in a receptor-mediated manner. Meanwhile, melatonin can also be soluble into water and lipid environments, thus easily diffusing through cell membranes and penetrating cellular compartments to work through non-receptor-mediated mechanisms (Mao et al., 2010; Liu et al., 2016; Asghari et al., 2017; Ferlazzo et al., 2020). Melatonin exerts oncostatic functions in numerous human malignancies, containing breast cancer, ovarian cancer, prostate cancer, skin cancer, and liver cancer, etc. (Zhao et al., 2019). Melatonin could suppress cancer progression through regulating cancer cell proliferation, migration, invasion, angiogenesis, apoptosis and cell cycle (Fernandez-Palanca et al., 2021). In particular, as a natural compound, melatonin intriguingly plays antithetical roles in normal cells and cancer cells. For example, melatonin increases the activity of SIRT1 and inhibits apoptosis by reducing the expression of Ac-FOXO1A, Ac-TP53, or Ac-BAX in normal cells. Conversely, SIRT1 is highly activated in cancer cells, and melatonin blocks SIRT1, thereby inhibiting cell proliferation and exerting a tumor suppressing effect (Mayo et al., 2017). However, due to the clinical complexity, the anticancer mechanisms of melatonin in cancer therapeutics are still largely less understood.

Alternative splicing (AS) is a crucial post-transcriptional mechanism to regulate gene expression patterns that allows a single gene to code for multiple transcript isoforms, thereby increasing the diversity and complexity of the transcriptome (Yuan et al., 2017; Hu et al., 2020). Alternative splicing plays an important role in tumorigenesis and cancer progression. Aberrant splicing could induce the production of noncanonical and cancer-specific mRNA transcripts, causing the inactivation of tumor suppressors or the activation of oncogenes (Chen et al., 2019). These RNA variants could be translated into distinct protein isoforms, and might be involved in different tumor biological functions, such as proliferation, apoptosis, angiogenesis, metabolism, stemness, drug-resistance and metastasis (Bonnal et al., 2020; Sciarrillo et al., 2020). Importantly, abnormal RNA alternative splicing can promote the development of HCC. For example, BIN1 generates a short isoform (BIN1-S, which lacks exon 12a) that exerts a tumor suppressing effect by inhibiting the binding of c-Myc to target gene promoter in the normal liver. However, upregulated NONO helps the oncogenic splicing switch of BIN1 from BIN1-S to BIN1-L (a long isoform, which contains exon 12) to promote carcinogenesis in HCC (Hu et al., 2020). Therefore, the study of molecular mechanisms of alternative splicing might provide novel therapeutics for liver cancer. However, whether alternative splicing is involved in melatonin-mediated inhibition of tumor progression is still largely unknown.

Here, we reported that melatonin can exert antitumor effects by regulating cancer-related splicing events in HCC. To systematically identify melatonin-regulated splicing events, we performed mRNA-seq analysis on HepG2 cells with melatonin treatment for 24 h and 48 h. Importantly, in addition to the regulation of gene expression, melatonin also modulated the occurrence of a wide range of splicing events. Briefly, we identified 391 overlapped differentially expressed genes and 335 overlapped AS events after melatonin treatment for 24 h and 48 h in HepG2. Our results showed that exon skipping is the predominant type of melatonin-induced alternative splicing events, and we identified the alternative splicing switches of *IKBKG*, *LPIN1*, *ITGA6*, *TERF1*, *KIF23*, *SIN3B*, which might regulate the mitotic cell cycle, kinase activity, GTPase regulator activity, cellular response to DNA damage stimulus, and histone modification functions. The regulatory effect of melatonin on alternative splicing is partly mediated by MT1. Moreover, melatonin could exert tumor suppressing effect by up-regulating the expression of the long isoform of *IKBKG*. Taken together, our study systematically identified melatonin-mediated alternative splicing events, which might provide a new avenue to interpret the tumor suppressing function of melatonin in HCC.

## Materials and methods

### Cell culture and reagents

Human HCC HepG2 cell line and Hep3B cell line were obtained from the American Type Culture Collection. HepG2 cell line and Hep3B cell line were maintained at standard culture conditions (37°C, 5% CO_2_) in MEM medium with 10% FBS (BI), Sodium Pyruvate (macgene, CC007), Nonessential Amino Acids Solution (macgene, CC25025). HEK-293T cells were maintained at standard culture conditions (37°C, 5% CO_2_) in DMEM medium with 10% FBS. NCI-H1299 cells were maintained at standard culture conditions (37°C, 5% CO_2_) in 1640 medium with 10% FBS. A549 cells were maintained at standard culture conditions (37°C, 5% CO_2_) in F12K medium with 10% FBS. Melatonin was purchased from Selleck (S1204).

### Plasmid constructions and generation of stable cell lines

To generate the mammalian expression plasmids pCDH-Flag-NEMO-L and pCDH-Flag-NEMO-S, human NEMO-L and NEMO-S cDNA were amplified by PCR and then cloned into lentivirus vector pCDH-CMV-MCS-EF1-Puro with N-terminal Flag tag with restriction enzymes Nhe I and Not I. shRNAs targeting MT1 were cloned into the pLKO.1. To stably overexpress NEMO-L/S or knockdown MT1 in HepG2 cells, lentiviral particles were produced by transient transfection of HEK-293T cells with pCDH-Flag-NEMO-L or pCDH-Flag-NEMO-S or pCDH-empty or plko.1-empty or plko.1-shMT1 vectors. Media contains lentivirus were used to infect HepG2 cells, followed by 4 μg/ml puromycin (Solarbio, P8230) selection for 5 days. The expression of transgenes was confirmed by western blots, semi-quantitative RT-PCR or RT-qPCR before further analysis.

### Western blot

Cells were washed twice with cold 1 × PBS and then lysed by RIPA lysis buffer containing 1 mM PMSF and 1 mM Cocktail. Cells were scraped off and collected into EP tubes, and centrifuged at 12,000 rpm for 15 min to remove cell debris. Equal amount of total protein was separated by 10% SDS-PAGE and transferred to nitrocellulose membrane, which were blocked with 5% fat-free milk and incubated with primary antibody at 4°C overnight. The following antibodies were used: Anti-Flag (Sigma, F1804), anti-GAPDH (Proteintech, 60004-1-Ig) and anti-NEMO (Proteintech, 18474-1-AP). After PBS washes, the membrane was incubated with secondary antibodies for 1 h at room temperature. Finally, bound antibodies were visualized with the ECL enhanced chemiluminescence regent Kit (NCM Biotech).

### RT-qPCR and semi-quantitative RT-PCR

Total RNAs were extracted from cells treated with or without melatonin using TRIzol reagent (Invitrogen) according to the manufacturer’s instructions. Genomic DNAs were removed and total RNA (2 μg) was reverse transcribed by the Thermo Scientific RevertAid First Strand cDNA Synthesis Kit (with DNase I). We performed RT-qPCR used MonAmp™ Taqman qPCR Mix (Monad) according to the manufacturer’s instructions. The expression level of targets was normalized to the endogenous expression of *GAPDH*. The cDNA was also used as the template for semi-quantitative RT-PCR. Products were separated on 3% agarose gels, and imaged were captured using a CCD camera (Tanon 2500R). The primers listed in Supplementary Table S1.

### RNA-seq analysis

HepG2 cells were treated with melatonin 1 mM or DMSO for 24 h and 48 h, and then extracted total RNAs using TRIzol and cleaned using RNAeasy Kit (Qiagen). The DNA was removed from total RNAs by digesting in column with RNase free DNase according to manufacturer’s instructions. Polyadenylated RNA were purified from total RNA by using Illumina TruSeq Total RNA Sample Prep kits. For discover splicing junctions, we mapped the paired-end sequences to human genome (hg38) using Map Splice 2.0.1.6 (default parameters). The level of gene expression was analyzed by the mapped reads with Cufflinks. We analyzed the changes of splicing isoforms using MISO package with annotation of all known alternative splicing events, and the results filtered according to the PSI values.

We preformed gene ontology analysis and KEGG analysis using metascape.org to search for enriched functions and pathways. The functionally correlated network of melatonin-regulated genes was analyzed by STRING database.

### IC50 measurement and growth curve assay

CCK8 assay was used to detect the IC50. Cells were seeded into 96-well plates (4000 cells/well) to culture overnight and then treated with different concentrations (0, 0.5, 1, 1.5, 2.5, 5, 7.5, 10 mM) of melatonin for 72 h. Then 10 µL/well CCK8 was added, allowing cells to continuously culture at 37°C for 2 h. Absorbance at 450 nm was measured using an ELISA reader (DNM-9602, Perlong). Dose-response curves were plotted to determine half maximal IC50 of melatonin using the GraphPad Prism. For growth curve assay, cells were seeded into 96-well plates (1000 cells/well) and cell viability was measured using CCK8 assay. Cell growth curves were determined by absorbance at 450 nm.

### Colony formation

Cells were seeded in 60-mm dishes (1500 cells per dish) and incubated at 37°C, 5% CO_2_ in humidified incubator for 15 days. Each treatment was carried out in triplicate. Colonies were fixed with 4% PFA and stained with crystal violet.

### Liver cancer tissue specimens

We collected fresh liver cancer tissues and adjacent normal tissues from patients with pathologically and clinically confirmed liver cancer. All human tumor tissues were obtained with written informed consent from patients or their guardians prior to participation in the study. The Institutional Review Board of the Dalian Medical University approved use of the tumor specimens in this study. All of tissue specimens were immediately frozen in liquid nitrogen and kept at −80°C until the extraction of RNA.

### Statistical analysis

Data was presented as mean ± SD. Statistical significance was determined by unpaired *t* test, one-way ANOVA or two-way ANOVA. Statistical analyses were performed using GraphPad Prism 7 (NS, not significant; *p < 0.05, **p < 0.01, ***p < 0.001, ****p < 0.0001).

## Results

### Melatonin inhibits HCC cell proliferation

**FIGURE 1:**
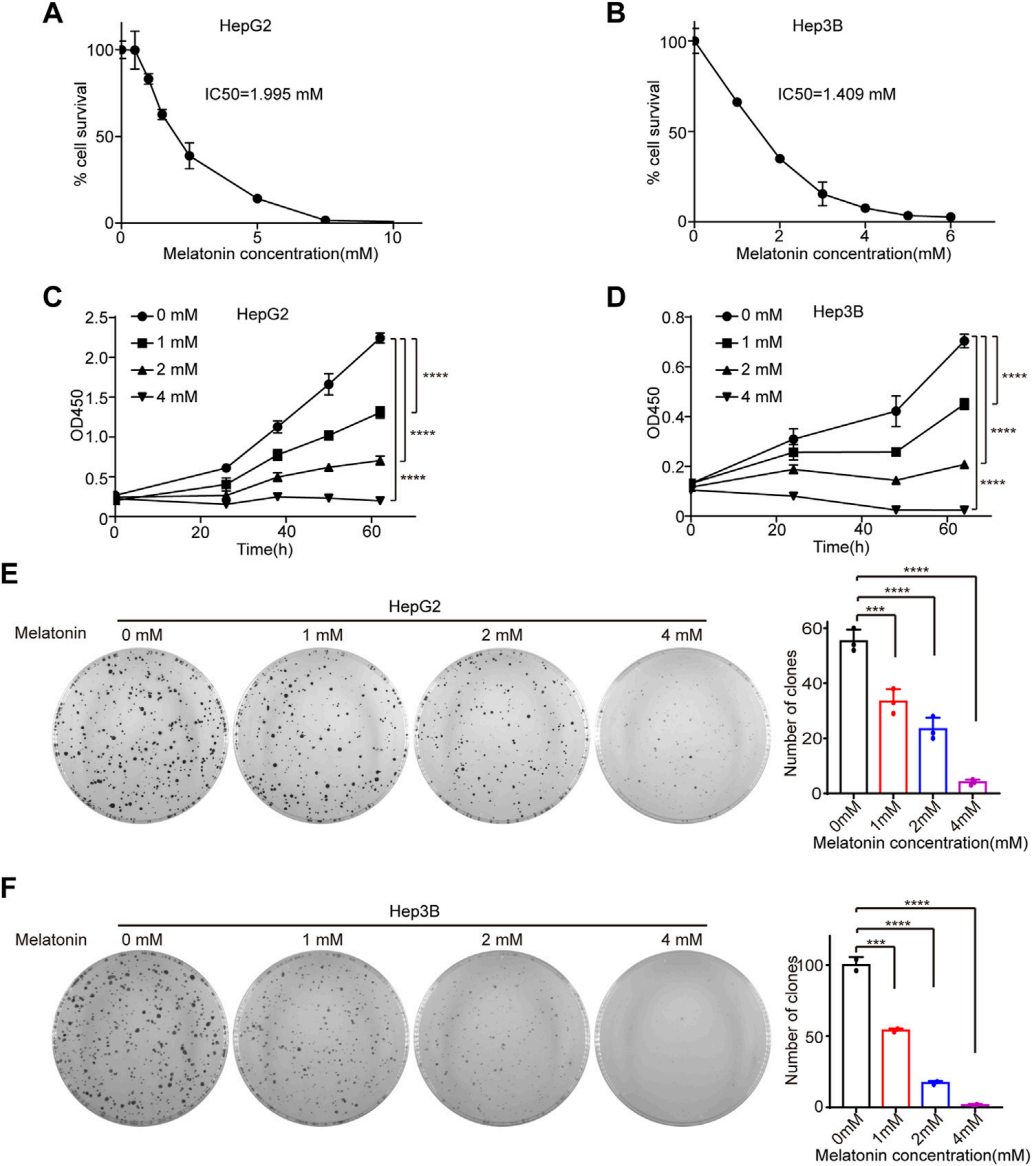
Melatonin inhibits cell proliferation. **(A)** HepG2 cells were treated with gradient concentrations of 0, 0.5, 1, 1.5, 2.5, 5, 7.5, 10 mM melatonin for 72 h, and cell viability was measured by CCK8 assay. **(B)** Hep3B cells were treated with gradient concentration of 0, 1, 2, 3, 4, 5, 6 mM melatonin for 72 h, and cell viability was measured by CCK8 assay. IC50 were calculated using GraphPad Prism. **(C,D)** The cell viability of HepG2 cells and Hep3B cells treated with gradient concentration of 0, 1, 2, 4 mM melatonin for 62 h, was measured by CCK8 assay at intervals, with *p* values calculated by two-way ANOVA. **(E,F)** The proliferation of HepG2 cells and Hep3B cells treated with gradient concentration of 0, 1, 2, 4 mM melatonin was examined with colony formation assay. Images of the whole plate are shown. The mean ± SD of relative colony numbers was plotted and *p* values calculated by one-way ANOVA.

To explore the role of melatonin in HCC, HepG2 and Hep3B cells were treated with different concentrations of melatonin for 72 h and cell viability was determined by cell counting kit-8 (CCK8). The half maximal inhibitory concentration (IC50) of melatonin in HepG2 and Hep3B cells were 1.995 mM and 1.409 mM, respectively (Figures 1A,B). Importantly, melatonin inhibits cell proliferation in a dose-dependent manner in HCC HepG2 and Hep3B cells as judged by CCK8 and colony formation assay (Figures 1C–F). The treatment of 1 mM of melatonin showed a significant inhibition of cell growth, whereas higher concentrations (2 and 4 mM) demonstrated a much stronger inhibitory effect in HepG2 and Hep3B cells (Figures 1C–F). Altogether, melatonin can inhibit the growth of HCC cells in a dose-dependent manner.

### Analysis of gene expression changes induced by melatonin

**FIGURE 2:**
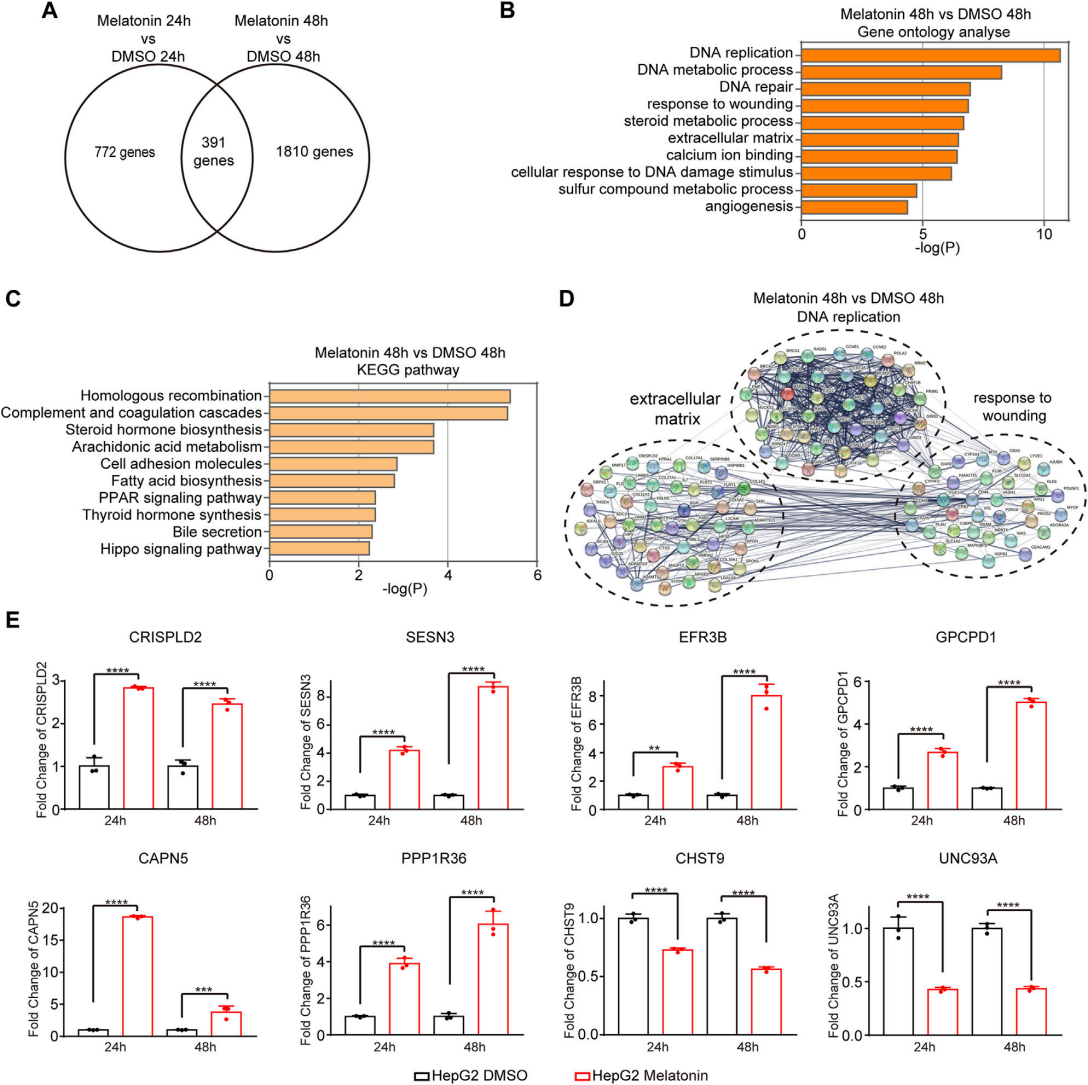
Analysis of gene expression changes induced by melatonin. **(A)** Venn diagram showed the overlapping genes differentially expressed in HepG2 cells treated with melatonin for 24 h and 48 h **(B)** Gene ontology analyses of gene expression events induced by melatonin treatment for 48 h. Fisher exact *p* values were plotted for each category. **(C)** KEGG pathway analyses of gene expression events induced by melatonin treatment for 48 h. **(D)** Functional association network of melatonin-induced gene expression changes. Genes in panel B were analyzed using the STRING database, and subgroups are marked according to their functions. **(E)** The identified gene expression changes were validated by RT-qPCR. The mean ± SD of relative fold changes was plotted (*n* = 3) with *p* values calculated by one-way ANOVA.

To further explore the molecular mechanism of melatonin-induced cell growth inhibitory, we performed high-throughput mRNA sequencing (mRNA-seq) with HepG2 cells treated with 1 mM melatonin for 24 h and 48 h separately. We identified 1163 genes and 2201 genes with significant expression change after 24 h and 48 h treatment with melatonin respectively as compared to controls (*p* < 0.05). Among which, 391 overlapping genes were differentially expressed in HepG2 cells treated with melatonin for 24 h and 48 h (Figure 2A). These genes are closely associated with DNA replication, DNA metabolic process, response to wounding, steroid metabolic process, extracellular matrix, calcium ion binding, cellular response to DNA damage stimulus, sulfur compound metabolic process and angiogenesis as judged by gene ontology analysis (Figure 2B, and Supplementary Figure S1A). In addition, those genes are enriched in homologous recombination, complement and coagulation cascades, steroid hormone biosynthesis, arachidonic acid metabolism, cell adhesion molecules, fatty acid biosynthesis, and PPAR signaling pathway as judged by KEGG analysis (Figure 2C, and Supplementary Figure S1B). Most of melatonin treatment for 48 h induced genes were functionally connected into a well linked interaction network that contains genes associated with DNA replication, response to wounding and extracellular matrix, as judged by the Search Tool for the Retrieval of Interacting Genes/Proteins (STRING) (Figure 2D). Meanwhile, many of those genes induced by melatonin treatment for 24 h were functionally correlated into a network that includes genes correlated with steroid metabolic process, response to wounding and extracellular matrix (Supplementary Figure S1C). Several target genes-induced by melatonin were randomly selected to further validate with quantitative real-time RT-PCR (RT-qPCR) in HepG2 cells treated with 1 mM melatonin for 24 h and 48 h, and the changes were consistent with the results obtained from mRNA-seq (Figure 2E). Among these genes, *CRISPLD2* and *SESN3* are involved in extracellular matrix. *EFR3B* and *GPCPD1* participate in regulating phospholipid metabolic process function. Taken together, melatonin can regulate the expression of cancer-related genes.

### Analysis of alternative splicing events induced by melatonin

**FIGURE 3:**
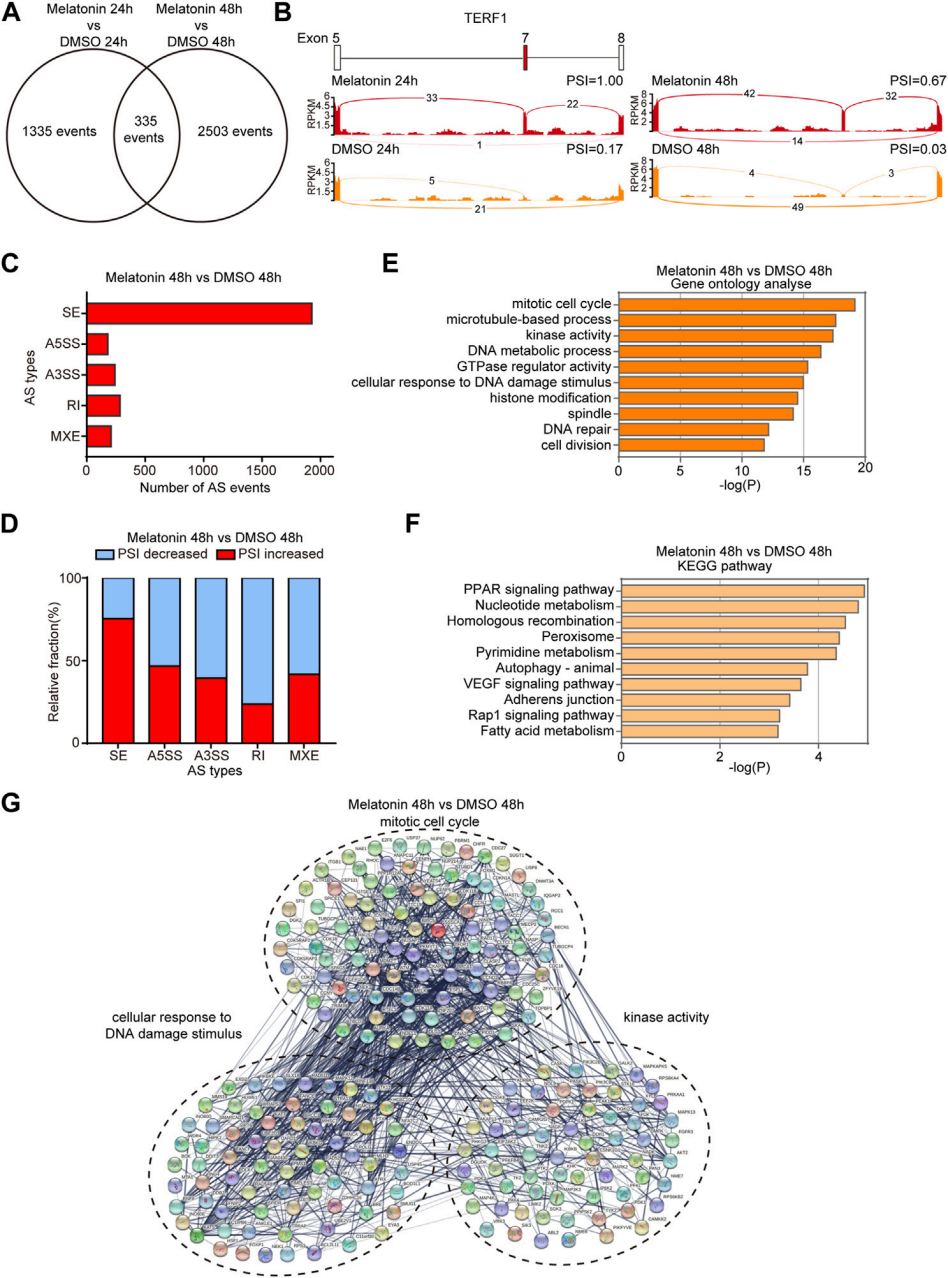
Analysis of alternative splicing events induced by melatonin. **(A)** Venn diagram showed the overlapping AS events differentially expressed in HepG2 cells treated with melatonin for 24 h and 48 h. **(B)** Example of alternative splicing affected by melatonin. Alternative splicing of *TERF1* was chosen to represent an increase of PSI, and numbers of exon junction reads were indicated. **(C)** Quantification of the different AS events affected by melatonin treatment for 48 h. **(D)** The relative fraction of each AS event positively or negatively induced by melatonin treatment for 48 h. **(E)** Gene ontology analyses of AS events regulated by melatonin treatment for 48 h. Fisher exact *p* values were plotted for each category. **(F)** Pathways were analyzed by KEGG pathway database for melatonin treatment 48 h induced AS events. **(G)** Functional association network of melatonin-induced AS events using the STRING database.

In order to explore the effect of melatonin on AS, we systematically analyzed mRNA-seq data to identify differentially changed AS events. We respectively identified 1670 and 2838 AS events with a significant change of percent-spliced-in (PSI) values (the change of PSI >0.15) after 24 h and 48 h treatment with melatonin compared to controls. 335 overlapping AS events were differentially expressed in HepG2 cells treated with melatonin for 24 h and 48 h (Figure 3A). Figure 3B shows the read tracks of one example. We found that melatonin can modulate various types of AS, including skipped exon (SE), alternative 5′ ss exon (A5E), alternative 3′ ss exon (A3E), retained intron (RI) and mutually exclusive exons (MXE) (Figure 3C and Supplementary Figure S2A). Subsequent analysis indicated that most of the SE events were positively regulated by melatonin (increased PSI value) while most of the RI events were negatively controlled (decreased PSI value) (Figure 3D and Supplementary Figure S2B).

We analyzed cellular functions of melatonin-induced AS events using gene ontology and found that these genes are associated with mitotic cell cycle, microtubule-based process, kinase activity, DNA metabolic process, GTPase regulator activity, cellular response to DNA damage stimulus, histone modification, spindle, DNA repair and cell division as judged by gene ontology analysis (Figure 3E and Supplementary Figure S2C). Meanwhile, the AS events are abundantly enriched in PPAR signaling pathway, nucleotide metabolism, homologous recombination, peroxisome and other pathways as judged by KEGG analysis (Figure 3F and Supplementary Figure S2D). In addition, we performed STRING analysis and found that many melatonin-regulated AS events were functionally connected into well linked interaction networks that contains genes associated with mitotic cell cycle, cellular response to DNA damage stimulus and kinase activity (Figures 3G, Supplementary Figure S2E). Taken together, these results suggest that the biological processes affected by melatonin are related to tumorigenesis and cancer progression.

### Alternative splicing switches induced by melatonin

**FIGURE 4:**
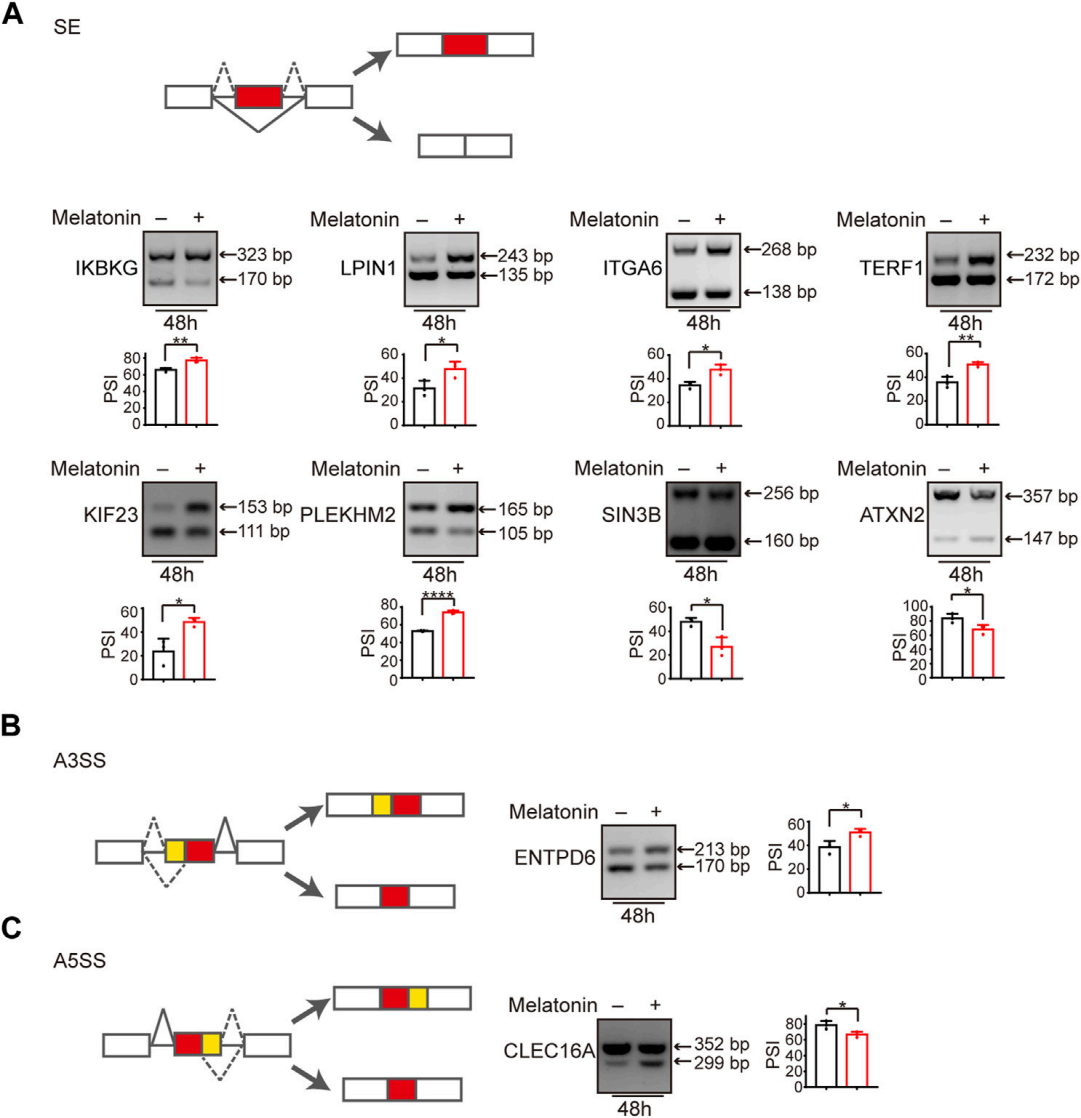
Alternative splicing switch induced by melatonin. **(A)** Exons skipping in *IKBKG*, *LPIN1*, *ITGA6*, *TERF1*, *KIF23*, *PLEKHM2*, *SIN3B*, and *ATXN2* were examined by semi-quantitative RT-PCR in HepG2 cells treated with 1 mM melatonin for 48 h. The mean ± SD of PSIs from three experiments were plotted and *p* values calculated by unpaired *t* test. **(B,C)** Alternative 3′ splice sites usage of *ENTPD6* and alternative 5′ splice sites usage of *CLEC16A* were examined by semi-quantitative RT-PCR in HepG2 cells treated with 1 mM melatonin for 48 h. The mean ± SD of PSIs from three experiments were plotted and *p* values calculated by unpaired *t* test.

We subsequently validated mRNA-seq results by measuring the splicing change of randomly chosen targets in HepG2 and Hep3B cells treated with melatonin by the semi-quantitative RT-PCR assay. Consistent with the results from mRNA-seq analysis, melatonin promoted exon 5 splicing of *IKBKG* (inhibitor of nuclear factor kappa-B kinase subunit gamma). Similarly, melatonin also stimulated the inclusion of exon 6 of *LPIN1* (phosphatidic acid phosphatase 1), exon 25 of *ITGA6* (Integrin alpha-6), exon 7 of *TERF1* (telomeric repeat-binding factor 1), exon 8 of *KIF23* (mitotic kinesin-like protein 1) and exon 7 of *PLEKHM2* (Pleckstrin homology domain-containing family M member 2). Meanwhile, melatonin could induce the skipping of exon 10 of *SIN3B* (histone deacetylase complex subunit) and exon 10 of *ATXN2* (spinocerebellar ataxia type 2 protein) (Figure 4A and Supplementary Figures S3,S4). In addition, other types of splicing events induced by melatonin were also validated. Briefly, melatonin promoted the upstream 3′ ss usage of *ENTPD6* (ectonucleoside triphosphate diphosphohydrolase 6) and inhibited the distal 5′ ss usage of *CLEC16A* (c-type lectin domain family 16 member A) (Figures 4B,C). Among these genes, *IKBKG* encodes nuclear factor κB essential modulator (NEMO), which acts as a tumor repressor in HCC. Moreover, NEMO protects the liver against chronic inflammation, progression of nonalcoholic steatohepatitis, and hepatocarcinogenesis (Luedde et al., 2007; Kondylis et al., 2017). *LPIN1* could regulate nuclear remodeling, mediating the effect of mTORC1 on SREBP pathway (Peterson et al., 2011). *SIN3B* is a transcription suppressor, which interacts with MXI1 to repress MYC responsive genes, thereby antagonizing MYC oncogenic activities (Alland et al., 1997). *SIN3B* also regulates cell cycle progression by repressing the expression of cell cycle inhibitor genes (Bowman et al., 2014). Taken together, melatonin could regulate a variety of splicing events in HCC.

## The regulatory effect of melatonin on alternative splicing is partially dependent on MT1

**FIGURE 5:**
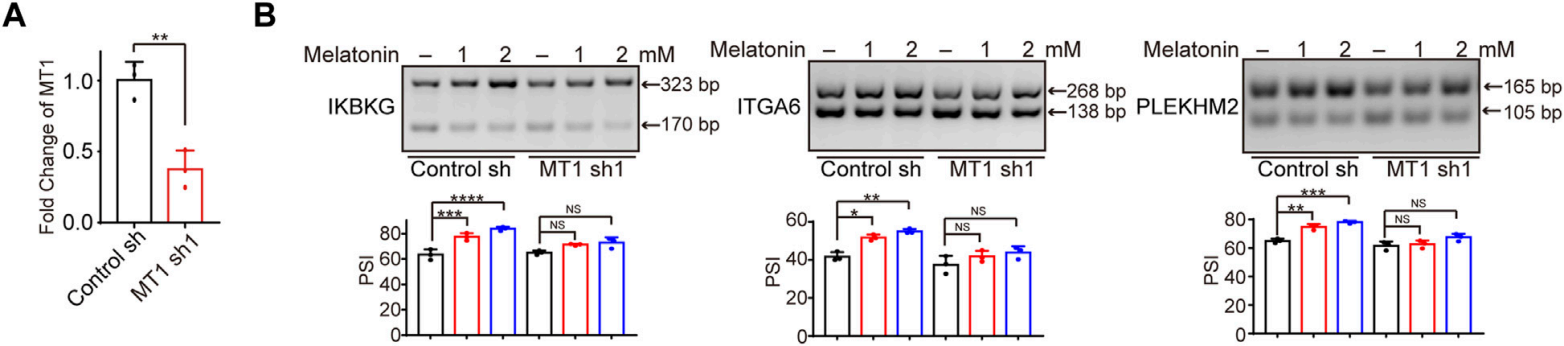
The regulatory effect of melatonin on alternative splicing is dependent on MT1. **(A)** The MT1 expression was validated by RT-qPCR. **(B)** Exons skipping of *IKBKG*, *ITGA6*, and *PLEKHM2* were examined by semi-quantitative RT-PCR in control and MT1-konckdown HepG2 cells treated with 1mM and 2 mM melatonin for 24 h. The mean ± SD of PSIs from three experiments were plotted and *p* values calculated by one-way ANOVA.

Previous study reported that melatonin exerted anti-tumor effect through interacting with melatonin receptors MT1 or MT2 (Mao et al., 2010; Liu et al., 2016; Asghari et al., 2017; Ferlazzo et al., 2020). Therefore, we investigated whether the regulation of alternative splicing by melatonin is dependent on melatonin receptors. Since MT2 is not expressed in HepG2 cells (Carbajo-Pescador et al., 2009; Carbajo-Pescador et al., 2011), we constructed HepG2 cells with MT1 stable depletion, and verified the knockdown efficiency by RT-qPCR (Figure 5A). Subsequently, we treated MT1-depleted and control HCC cells with different concentrations of melatonin for 24 h, and examined splicing switches using semi-quantitative RT-PCR. Importantly, we found that melatonin-induced splicing switches are at least partially dependent on the status of MT1. We revealed that melatonin promoted the production of the long-isoforms of *IKBKG*, *ITGA6*, and *PLEKHM2* in a dose-dependent manner, while the regulatory effect of melatonin was significantly attenuated in MT1-depleted HepG2 cells (Figure 5B). Overall, the regulatory effect of melatonin on alternative splicing in HCC cells is at least partly modulated by MT1.

### *IKBKG* splicing switch participated in melatonin-induced HCC inhibition

**FIGURE 6:**
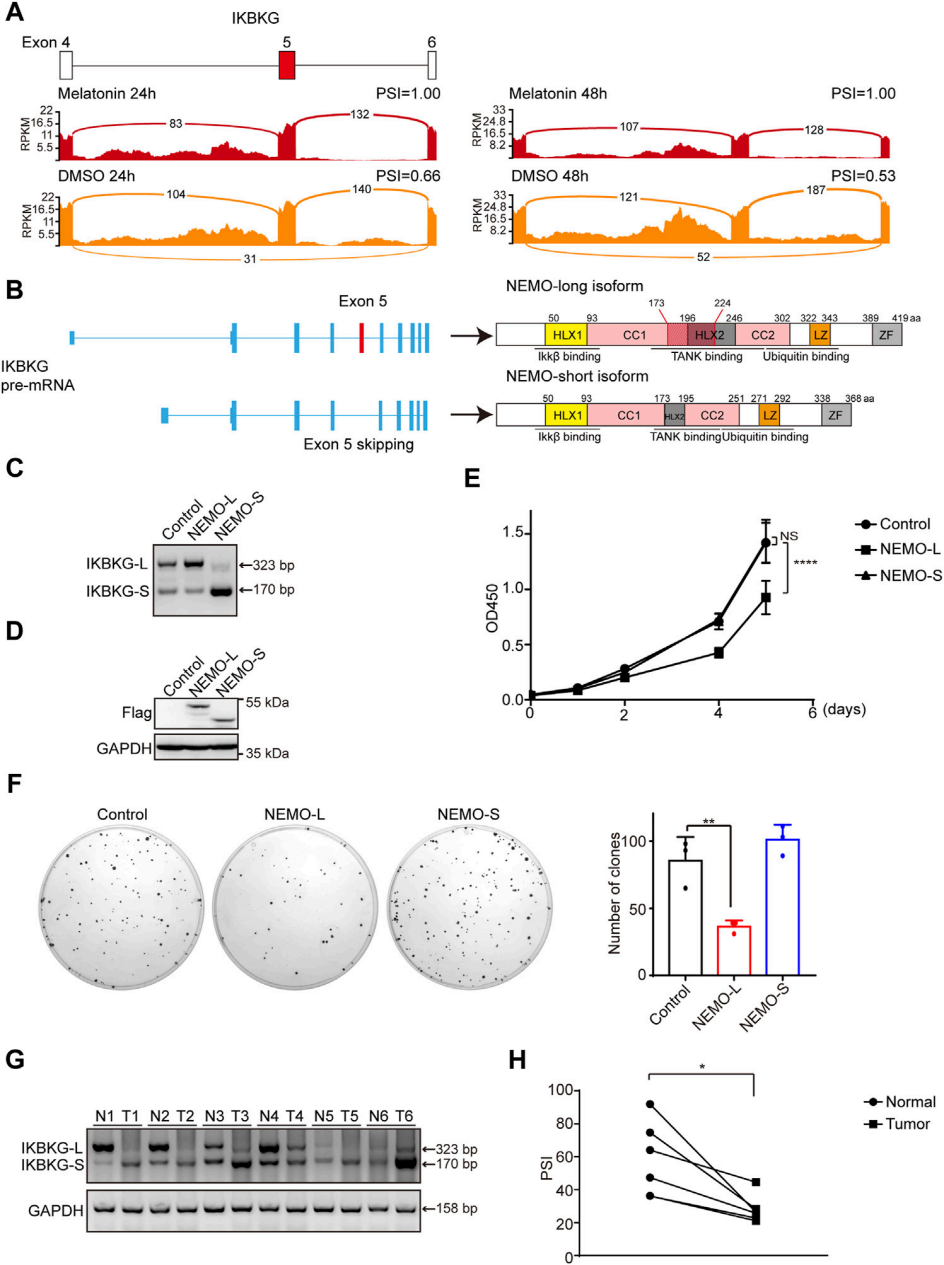
NEMO-L inhibits cell proliferation. **(A)** Alternative exon of *IKBKG* affected by melatonin. Numbers of exon junction reads and PSI were indicated. **(B)** Schematics of human *IKBKG* (NEMO) pre-mRNA and protein. NEMO-long isoform included the exon 5, while NEMO-short isoform skipped the exon 5. **(C,D)** HepG2 cells with stable expression of Flag-tagged NEMO-L or NEMO-S were constructed. RNA levels of *IKBKG* were examined by semi-quantitative RT-PCR **(C)**. Protein levels of exogenous NEMO were confirmed by western blot analysis using anti-Flag antibodies **(D)**. **(E)** Cell proliferation of HepG2 cells stably expressing NEMO-L or NEMO-S were analyzed by CCK8 assay at intervals, with *p* values calculated by two-way ANOVA. **(F)** Colony formation assays of HepG2 cells stably expressing NEMO-L or NEMO-S. Images of the whole plate are shown. The mean ± SD of relative colony numbers was plotted (*n* = 3) and *p* values calculated by one-way ANOVA. **(G,H)** Alternative splicing of *IKBKG* were measured in six paired liver cancer tissues and adjacent normal tissues by semi-quantitative RT-PCR. PSI values were plotted. * Indicated *p* < 0.05.

We have demonstrated that melatonin promoted the inclusion of exon 5 of *IKBKG*, resulting in an increase of full length of *IKBKG* (Figure 6A). In addition to liver cancer, we examined the splicing change of *IKBKG* upon melatonin treatment in NCI-H1299 and A549 lung cancer cells, and human embryonic kidney HEK-293T cells. We found that melatonin could affect the splicing of *IKBKG* in multiple cell lines (Supplementary Figure S5A). Importantly, *IKBKG* encoding protein NEMO/IKK-γ, together with IKK-α and IKK-β to form IKK complex, thereby regulating the activity of NF-κB. Previously, NEMO was identified as a tumor suppressor in liver by conditional hepatocyte-specific deletion of NEMO in mice (Luedde et al., 2007; Seki and Brenner, 2007; Kondylis et al., 2015; Mossanen et al., 2019). Moreover, NEMO expression was down-regulated in HCC as compared to their surrounding non-neoplastic liver tissues (Aigelsreiter et al., 2012). Therefore, we further performed western blot assay and validated that melatonin could increase the protein level of long isoform of NEMO (Supplementary Figure S5B). Next, we sought to investigate the role of the long isoform (NEMO-L) and the short isoform (NEMO-S) of *IKBKG* in HCC progression. NEMO-L isoform is the full-length transcript, whereas NEMO-S isoform lacks the exon 5 that encodes the domain associating with TANK (Figure 6B). In order to explore the effects of two NEMO isoforms on cell proliferation, we stably overexpressed NEMO-L or NEMO-S in HepG2 cells respectively. The expression of NEMO-L and NEMO-S were verified at both RNA and protein levels using semi-quantitative RT-PCR and western blot assays (Figures 6C,D). We found that HepG2 cells expressing NEMO-L grew much slower as compared to control cells, as well as HepG2 cells expressing NEMO-S as judged by CCK8 and colony formation assays (Figures 6E,F). These results suggested that NEMO-L, the full-length isoform, could inhibit HCC cell proliferation. Due to lack of exon 5, NEMO-S lost the ability to suppress HCC cell proliferation.

To further investigate the clinical significance of *IKBKG* splicing in cancer patients, we examined the relative levels of two *IKBKG* isoforms in six pairs of liver cancer samples and adjacent normal tissues. Relative mRNA levels of *IKBKG*-L were significantly decreased in six liver cancer samples compared with paired normal tissues (Figures 6G,H), indicating that despite the apparent heterogeneity of different tumor samples, the expression of *IKBKG*-L is generally reduced. Taken together, our results indicate that melatonin suppresses HCC progression through mediating *IKBKG* splicing.

## Discussion

Melatonin is a natural indole amine that is mainly produced by the pineal gland in human body. Melatonin has various functions, including the regulation of antioxidant and circadian rhythm. Numerous studies have shown that melatonin has significant anti-tumor activity in multiple cancers, including HCC, breast cancer, colorectal cancer, non-small lung cancer, and melanoma. Moreover, the tumor suppressing effect of melatonin is achieved by regulating various physiological functions, such as tumor proliferation, angiogenesis, metastasis, apoptosis, metabolism and immune escape (Talib, 2018; Talib et al., 2021). For example, melatonin and peroxisome proliferator-activated receptors (PPARγ) agonists synergistically induce apoptosis in breast cancer cells (Korkmaz et al., 2009). Melatonin inhibits proliferation of lung cancer cells by enhancing mitochondrial energy metabolism and reversed the Warburg effect (Chen et al., 2021). In addition, melatonin suppresses the migration and invasion of HCC cells by reducing the expression of VEGF and HIF1α (Colombo et al., 2016). Accumulating evidence suggests that melatonin has great potential in cancer therapy.

Alternative splicing of pre-mRNA is recognized as a key driver of proteomic diversity in human, by which a single gene can produce multiple isoforms with different or even opposite functions. For example, the long isoform of Bcl-x (Bcl-xL) plays an anti-apoptotic role, while its short isoform Bcl-xS functions as a pro-apoptotic factor (Wang et al., 2014). Alternative splicing can be deregulated in cancer, leading to the generation of aberrant splicing variants. These aberrant splicing variants can regulate tumorigenesis and cancer progression (Wang and Aifantis, 2020; Kitamura and Nimura, 2021). Accumulating evidence suggests that aberrant splicing variants confer therapeutic drug resistance in cancer (Sciarrillo et al., 2020). In our study, we demonstrated that melatonin exerts antitumor effects by regulating alternative splicing of many cancer-related genes. Specifically, melatonin could shift the splicing of *IKBKG* to increase the production of NEMO long isoform, thereby inhibiting cancer cell proliferation. This might be because that the full-length isoform of NEMO prevents RIPK1 activation and subsequent apoptosis by NF-κB-dependent or -independent functions, suppressing HCC progression (Kondylis et al., 2017). NEMO-S, the short isoform with the skipping of exon 5, impairs the TANK binding domain and cannot inhibit cancer cell proliferation. Our study provides a novel mechanism for melatonin to suppress cancer progression by regulating the alternative splicing of cancer-related genes.

In addition to the regulation of NEMO splicing, melatonin might also inhibit cancer progression through modulating the splicing of some other cancer-related genes. Using mRNA-seq analysis, we found that melatonin can regulate some splicing events related to mitotic cell cycle, kinase activity, DNA metabolic process, and GTPase regulator activity functions. For example, melatonin can regulate the splicing of *LPIN1* and *KIF23*, whose function is related to mitotic cell cycle. Taken together, melatonin may offer new opportunities for cancer therapeutics by regulating RNA alternative splicing.

## Data Availability

The datasets presented in this study can be found in online repositories. The names of the repository/repositories and accession numbers can be found below: https://www.ncbi.nlm.nih.gov/geo/, GSE210034.

## References

[B1] Aigelsreiter A. Haybaeck J. Schauer S. Kiesslich T. Bettermann K. Griessbacher A. (2012). NEMO expression in human hepatocellular carcinoma and its association with clinical outcome. Hum. Pathol. 43, 1012–1019. 10.1016/j.humpath.2011.08.009 22176836

[B2] Alland L. Muhle R. Hou H. Jr. Potes J. Chin L. Schreiber-Agus N. (1997). Role for N-CoR and histone deacetylase in Sin3-mediated transcriptional repression. Nature 387, 49–55. 10.1038/387049a0 9139821

[B3] Asghari M. H. Moloudizargari M. Bahadar H. Abdollahi M. (2017). A review of the protective effect of melatonin in pesticide-induced toxicity. Expert Opin. Drug Metab. Toxicol. 13, 545–554. 10.1080/17425255.2016.1214712 27434705

[B4] Bonnal S. C. Lopez-Oreja I. Valcarcel J. (2020). Roles and mechanisms of alternative splicing in cancer - implications for care. Nat. Rev. Clin. Oncol. 17, 457–474. 10.1038/s41571-020-0350-x 32303702

[B5] Bowman C. J. Ayer D. E. Dynlacht B. D. (2014). Foxk proteins repress the initiation of starvation-induced atrophy and autophagy programs. Nat. Cell Biol. 16, 1202–1214. 10.1038/ncb3062 25402684 PMC4250422

[B6] Carbajo-Pescador S. Garcia-Palomo A. Martin-Renedo J. Piva M. Gonzalez-Gallego J. Mauriz J. L. (2011). Melatonin modulation of intracellular signaling pathways in hepatocarcinoma HepG2 cell line: Role of the MT1 receptor. J. Pineal Res. 51, 463–471. 10.1111/j.1600-079X.2011.00910.x 21718361

[B7] Carbajo-Pescador S. Martin-Renedo J. Garcia-Palomo A. Tunon M. J. Mauriz J. L. Gonzalez-Gallego J. (2009). Changes in the expression of melatonin receptors induced by melatonin treatment in hepatocarcinoma HepG2 cells. J. Pineal Res. 47, 330–338. 10.1111/j.1600-079X.2009.00719.x 19817970

[B8] Chen H. Gao F. He M. Ding X. F. Wong A. M. Sze S. C. (2019). Long-read RNA sequencing identifies alternative splice variants in hepatocellular carcinoma and tumor-specific isoforms. Hepatology 70, 1011–1025. 10.1002/hep.30500 30637779 PMC6766942

[B9] Chen X. Hao B. Li D. Reiter R. J. Bai Y. Abay B. (2021). Melatonin inhibits lung cancer development by reversing the Warburg effect via stimulating the SIRT3/PDH axis. J. Pineal Res. 71, e12755. 10.1111/jpi.12755 34214200

[B10] Colombo J. Maciel J. M. Ferreira L. C. Rf D. A. S. Zuccari D. A. (2016). Effects of melatonin on HIF-1α and VEGF expression and on the invasive properties of hepatocarcinoma cells. Oncol. Lett. 12, 231–237. 10.3892/ol.2016.4605 27347130 PMC4907066

[B11] Ferlazzo N. Andolina G. Cannata A. Costanzo M. G. Rizzo V. Curro M. (2020). Is melatonin the cornucopia of the 21st century? Antioxidants (Basel) 9, E1088. 10.3390/antiox9111088 33167396 PMC7694322

[B12] Fernandez-Palanca P. Mendez-Blanco C. Fondevila F. Tunon M. J. Reiter R. J. Mauriz J. L. (2021). Melatonin as an antitumor agent against liver cancer: An updated systematic review. Antioxidants (Basel) 10, 103. 10.3390/antiox10010103 33445767 PMC7828223

[B13] Hu Z. Dong L. Li S. Li Z. Qiao Y. Li Y. (2020). Splicing regulator p54(nrb)/Non-POU domain-containing octamer-binding protein enhances carcinogenesis through oncogenic isoform switch of MYC box-dependent interacting protein 1 in hepatocellular carcinoma. Hepatology 72, 548–568. 10.1002/hep.31062 31815296

[B14] Huang A. Yang X. R. Chung W. Y. Dennison A. R. Zhou J. (2020). Targeted therapy for hepatocellular carcinoma. Signal Transduct. Target. Ther. 5, 146. 10.1038/s41392-020-00264-x 32782275 PMC7419547

[B15] Kitamura K. Nimura K. (2021). Regulation of RNA splicing: Aberrant splicing regulation and therapeutic targets in cancer. Cells, 2021 923. 10.3390/cells10040923 PMC8073995 33923658

[B16] Kondylis V. Kumari S. Vlantis K. Pasparakis M. (2017). The interplay of IKK, NF-κB and RIPK1 signaling in the regulation of cell death, tissue homeostasis and inflammation. Immunol. Rev. 277, 113–127. 10.1111/imr.12550 28462531

[B17] Kondylis V. Polykratis A. Ehlken H. Ochoa-Callejero L. Straub B. K. Krishna-Subramanian S. (2015). NEMO prevents steatohepatitis and hepatocellular carcinoma by inhibiting RIPK1 kinase activity-mediated hepatocyte apoptosis. Cancer Cell 28, 582–598. 10.1016/j.ccell.2015.10.001 26555174 PMC4644221

[B18] Korkmaz A. Tamura H. Manchester L. C. Ogden G. B. Tan D. X. Reiter R. J. (2009). Combination of melatonin and a peroxisome proliferator-activated receptor-gamma agonist induces apoptosis in a breast cancer cell line. J. Pineal Res. 46, 115–116. 10.1111/j.1600-079X.2008.00635.x 18798787

[B19] Liu J. Clough S. J. Hutchinson A. J. Adamah-Biassi E. B. Popovska-Gorevski M. Dubocovich M. L. (2016). MT1 and MT2 melatonin receptors: A therapeutic perspective. Annu. Rev. Pharmacol. Toxicol. 56, 361–383. 10.1146/annurev-pharmtox-010814-124742 26514204 PMC5091650

[B20] Liu X. Qin S. (2019). Immune checkpoint inhibitors in hepatocellular carcinoma: Opportunities and challenges. Oncologist 24, S3–S10. 10.1634/theoncologist.2019-IO-S1-s01 30819826 PMC6394775

[B21] Llovet J. M. Zucman-Rossi J. Pikarsky E. Sangro B. Schwartz M. Sherman M. (2016). Hepatocellular carcinoma. Nat. Rev. Dis. Prim. 2, 16018. 10.1038/nrdp.2016.18 27158749

[B22] Luedde T. Beraza N. Kotsikoris V. van Loo G. Nenci A. De Vos R. (2007). Deletion of NEMO/IKKgamma in liver parenchymal cells causes steatohepatitis and hepatocellular carcinoma. Cancer Cell 11, 119–132. 10.1016/j.ccr.2006.12.016 17292824

[B23] Mao L. Cheng Q. Guardiola-Lemaitre B. Schuster-Klein C. Dong C. Lai L. (2010). *In vitro* and *in vivo* antitumor activity of melatonin receptor agonists. J. Pineal Res. 49, 210–221. 10.1111/j.1600-079X.2010.00781.x 20609073

[B24] Mayo J. C. Sainz R. M. Gonzalez Menendez P. Cepas V. Tan D. X. Reiter R. J. (2017). Melatonin and sirtuins: A "not-so unexpected" relationship. J. Pineal Res. 62, e12391. 10.1111/jpi.12391 28109165

[B25] Montella L. Palmieri G. Addeo R. Del Prete S. (2016). Hepatocellular carcinoma: Will novel targeted drugs really impact the next future? World J. Gastroenterol. 22, 6114–6126. 10.3748/wjg.v22.i27.6114 27468204 PMC4945973

[B26] Mossanen J. C. Kohlhepp M. Wehr A. Krenkel O. Liepelt A. Roeth A. A. (2019). CXCR6 inhibits hepatocarcinogenesis by promoting natural killer T- and CD4(+) T-cell-dependent control of senescence. Gastroenterology 156, 1877–1889. 10.1053/j.gastro.2019.01.247 30710528

[B27] Peterson T. R. Sengupta S. S. Harris T. E. Carmack A. E. Kang S. A. Balderas E. (2011). mTOR complex 1 regulates lipin 1 localization to control the SREBP pathway. Cell 146, 408–420. 10.1016/j.cell.2011.06.034 21816276 PMC3336367

[B28] Sciarrillo R. Wojtuszkiewicz A. Assaraf Y. G. Jansen G. Kaspers G. J. L. Giovannetti E. (2020). The role of alternative splicing in cancer: From oncogenesis to drug resistance. Drug resist. updat. 53, 100728. 10.1016/j.drup.2020.100728 33070093

[B29] Seki E. Brenner D. A. (2007). The role of NF-kappaB in hepatocarcinogenesis: Promoter or suppressor? J. Hepatol. 47, 307–309. 10.1016/j.jhep.2007.05.006 17566588 PMC2739234

[B30] Sung H. Ferlay J. Siegel R. L. Laversanne M. Soerjomataram I. Jemal A. (2021). Global cancer statistics 2020: GLOBOCAN estimates of incidence and mortality worldwide for 36 cancers in 185 countries. Ca. A Cancer J. Clin. 71, 209–249. 10.3322/caac.21660 33538338

[B31] Talib W. H. Alsayed A. R. Abuawad A. Daoud S. Mahmod A. I. (2021). Melatonin in cancer treatment: Current knowledge and future opportunities. Molecules 26, 2506. 10.3390/molecules26092506 33923028 PMC8123278

[B32] Talib W. H. (2018). Melatonin and cancer hallmarks. Molecules 2018, E518. 10.3390/molecules23030518 PMC6017729 29495398

[B33] Tang W. Chen Z. Zhang W. Cheng Y. Zhang B. Wu F. (2020). The mechanisms of sorafenib resistance in hepatocellular carcinoma: Theoretical basis and therapeutic aspects. Signal Transduct. Target. Ther. 5, 87. 10.1038/s41392-020-0187-x 32532960 PMC7292831

[B34] Wang E. Aifantis I. (2020). RNA splicing and cancer. Trends Cancer 6, 631–644. 10.1016/j.trecan.2020.04.011 32434734

[B35] Wang Y. Chen D. Qian H. Tsai Y. S. Shao S. Liu Q. (2014). The splicing factor RBM4 controls apoptosis, proliferation, and migration to suppress tumor progression. Cancer Cell 26, 374–389. 10.1016/j.ccr.2014.07.010 25203323 PMC4159621

[B36] Yang J. D. Hainaut P. Gores G. J. Amadou A. Plymoth A. Roberts L. R. (2019). A global view of hepatocellular carcinoma: Trends, risk, prevention and management. Nat. Rev. Gastroenterol. Hepatol. 16, 589–604. 10.1038/s41575-019-0186-y 31439937 PMC6813818

[B37] Yuan J. H. Liu X. N. Wang T. T. Pan W. Tao Q. F. Zhou W. P. (2017). The MBNL3 splicing factor promotes hepatocellular carcinoma by increasing PXN expression through the alternative splicing of lncRNA-PXN-AS1. Nat. Cell Biol. 19, 820–832. 10.1038/ncb3538 28553938

[B38] Zhao D. Yu Y. Shen Y. Liu Q. Zhao Z. Sharma R. (2019). Melatonin Synthesis and function: Evolutionary history in animals and plants. Front. Endocrinol. 10, 249. 10.3389/fendo.2019.00249 PMC6481276 31057485

