# Supplementary material for: Melatonin inhibits HCC progression through regulating the alternative splicing of NEMO
Source: Front Pharmacol. 2022 Sep 26;13:1007006. doi: 10.3389/fphar.2022.1007006 (PMC9548564; doi:10.3389/fphar.2022.1007006)
Supplement: Supplementary file 1 [file DataSheet1.PDF]

Supplementary Figures

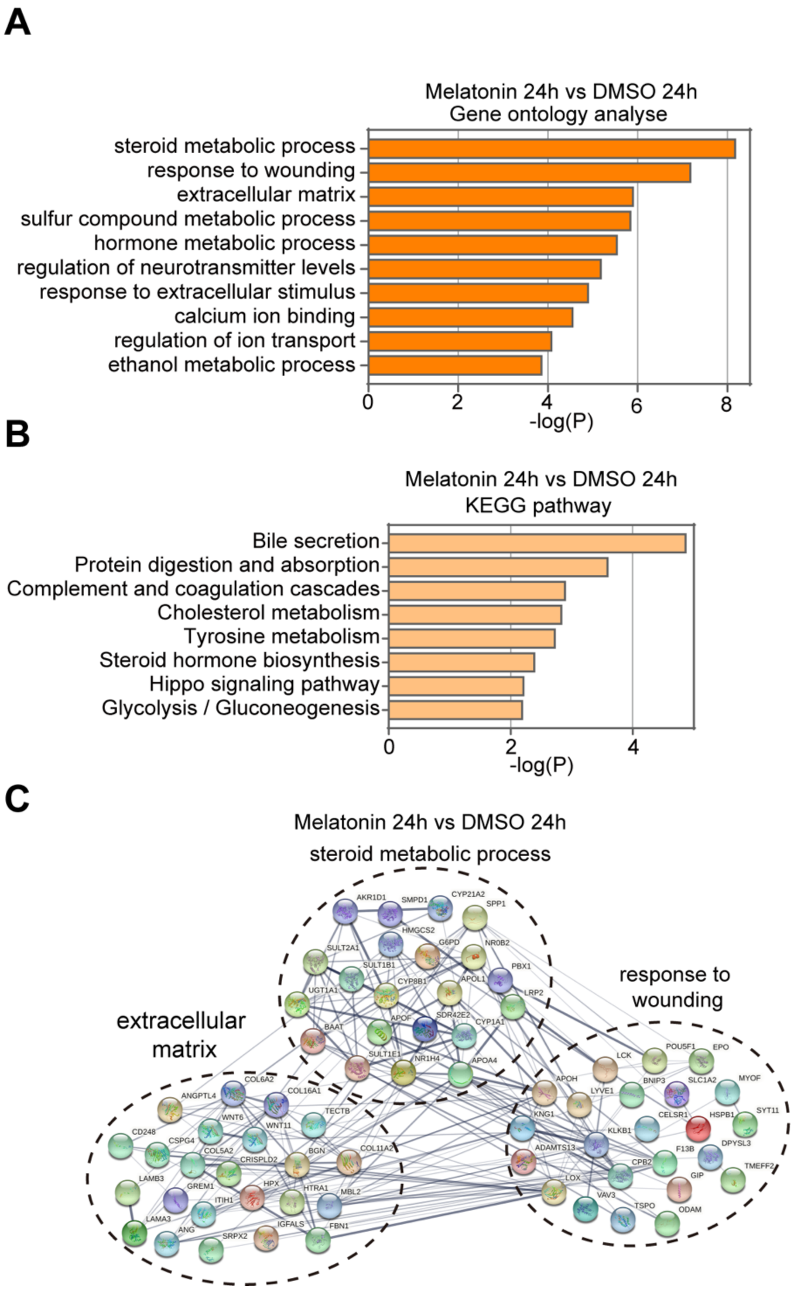

Fig. S1 Analysis of gene expression changes induced by melatonin. (A) Gene ontology analyses of gene expression events induced by melatonin for 24h. Fisher exact *p* values were plotted for each category. (B) Pathways were analyzed by KEGG pathway database for melatonin treatment for 24h induced gene expression changes. (C) Functional association network of melatonin-induced gene expression changes. Genes in panel A were analyzed using the STRING database, and subgroups are marked according to their functions.

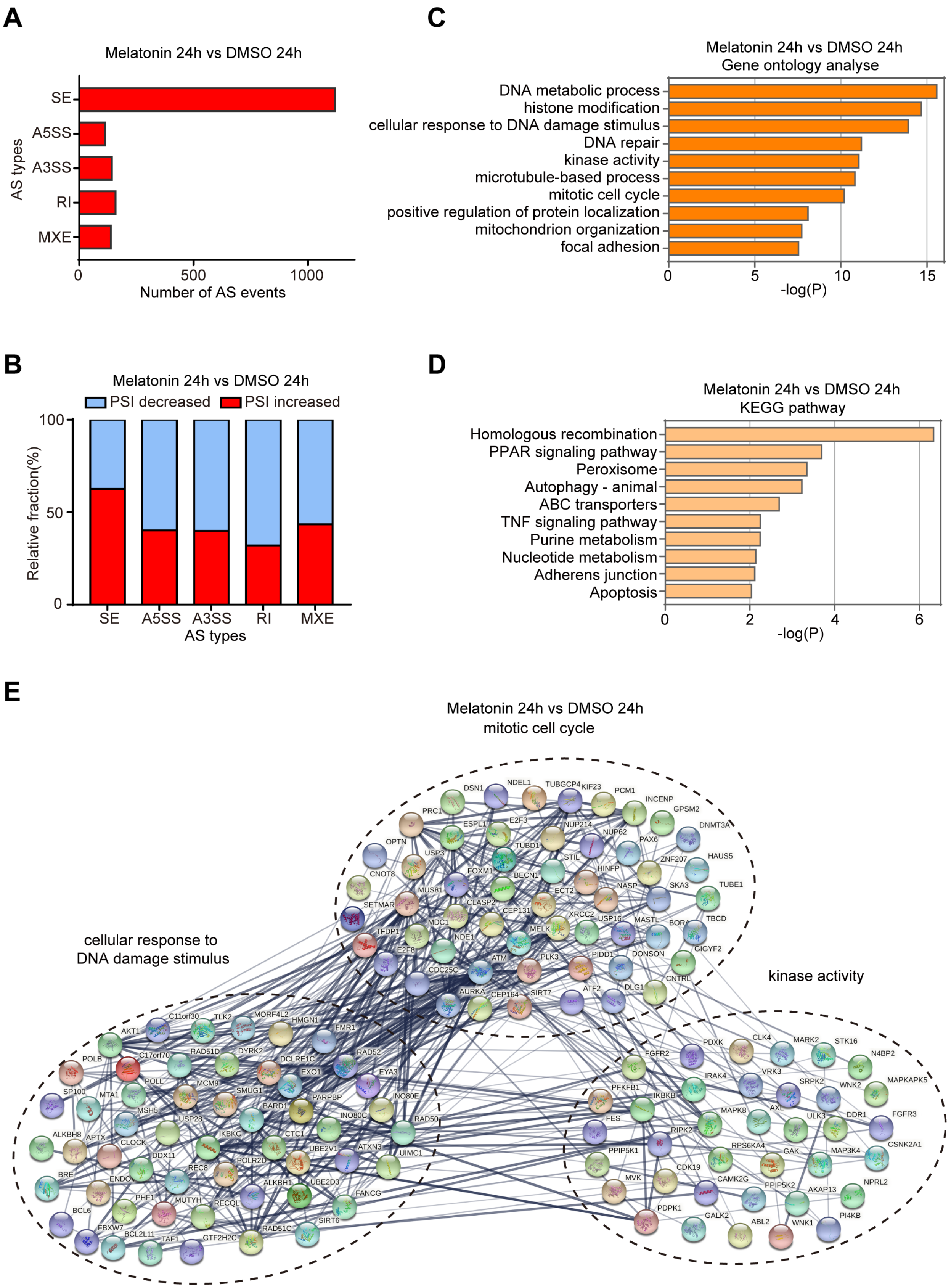

A
Melatonin 24h vs DMSO 24h
SE
A5SS
A3SS
RI
MXE
AS types
0
500
1000
Number of AS events
B
Melatonin 24h vs DMSO 24h
PSI decreased
PSI increased
100
50
0
Relative fraction(%)
SE
A5SS
A3SS
RI
MXE
AS types
C
Melatonin 24h vs DMSO 24h
Gene ontology analyse
DNA metabolic process
histone modification
cellular response to DNA damage stimulus
DNA repair
kinase activity
microtubule-based process
mitotic cell cycle
positive regulation of protein localization
mitochondrion organization
focal adhesion
0
5
10
15
-log(P)
D
Melatonin 24h vs DMSO 24h
KEGG pathway
Homologous recombination
PPAR signaling pathway
Peroxisome
Autophagy - animal
ABC transporters
TNF signaling pathway
Purine metabolism
Nucleotide metabolism
Adherens junction
Apoptosis
0
2
4
6
-log(P)
E
Melatonin 24h vs DMSO 24h
mitotic cell cycle
cellular response to
DNA damage stimulus
kinase activity

Fig. S2 Analysis of alternative splicing events induced by melatonin. (A) Quantification of the different AS events affected by melatonin treatment for 24 h. (B) The relative fraction of each AS event positively or negatively induced by melatonin treatment for 24 h. (C) Gene ontology analyses of AS events regulated by melatonin treatment for 24 h. Fisher exact *p* values were plotted for each category. (D) Pathways were analyzed by KEGG pathway database for melatonin treatment 24h induced AS events. (E) Functional association network of melatonin-induced AS events using the STRING database.

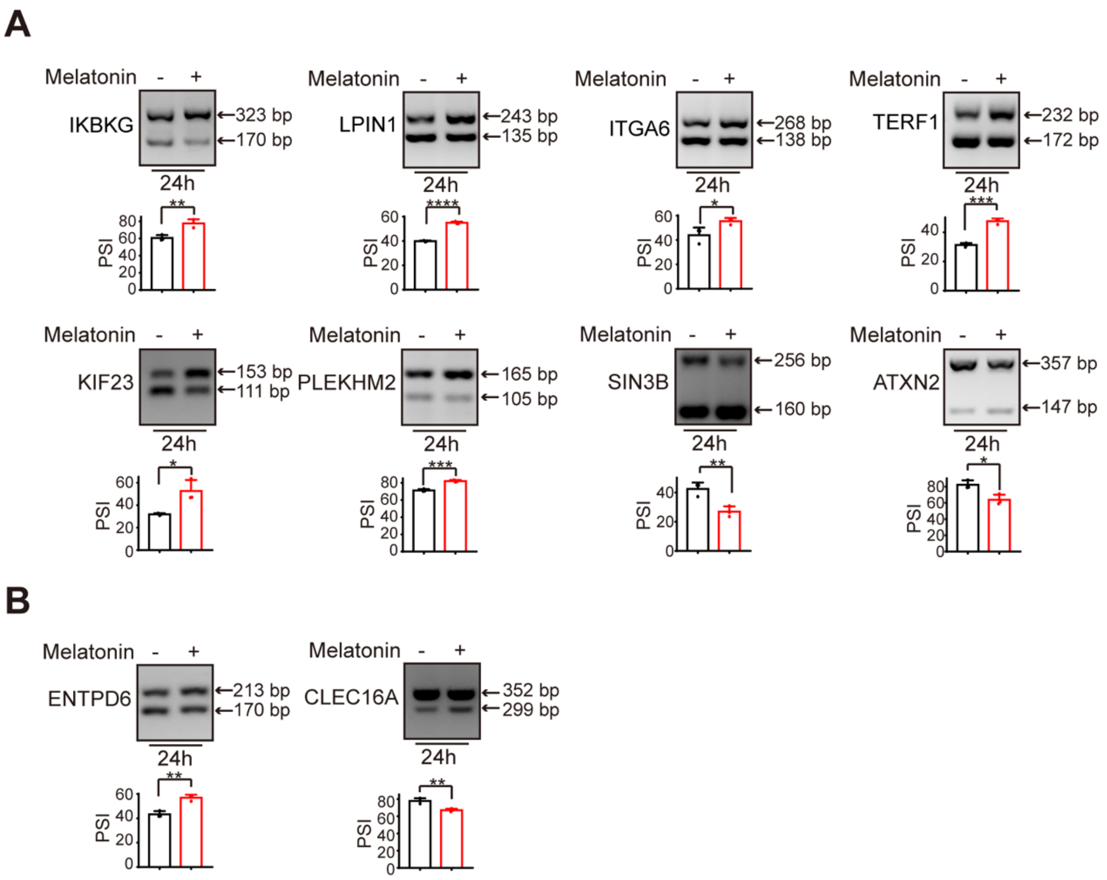

Fig. S3 Alternative splicing switch induced by melatonin. (A) Exons skipping in *IKBKG*, *LPIN1*, *ITGA6*, *TERF1*, *KIF23*, *PLEKHM2*, *SIN3B*, and *ATXN2* were examined by semi-quantitative RT-PCR in HepG2 cells treated with 1mM melatonin for 24h. The mean ± SD of PSIs from three experiments were plotted and *p* values calculated by unpaired t test. (B) Alternative 3′ splice sites usage of *ENTPD6* and alternative 5′ splice sites usage of *CLEC16A* were examined by semi-quantitative RT-PCR in HepG2 cells treated with 1mM melatonin for 24h. The mean ± SD of PSIs from three experiments were plotted and *p* values calculated by unpaired t test.

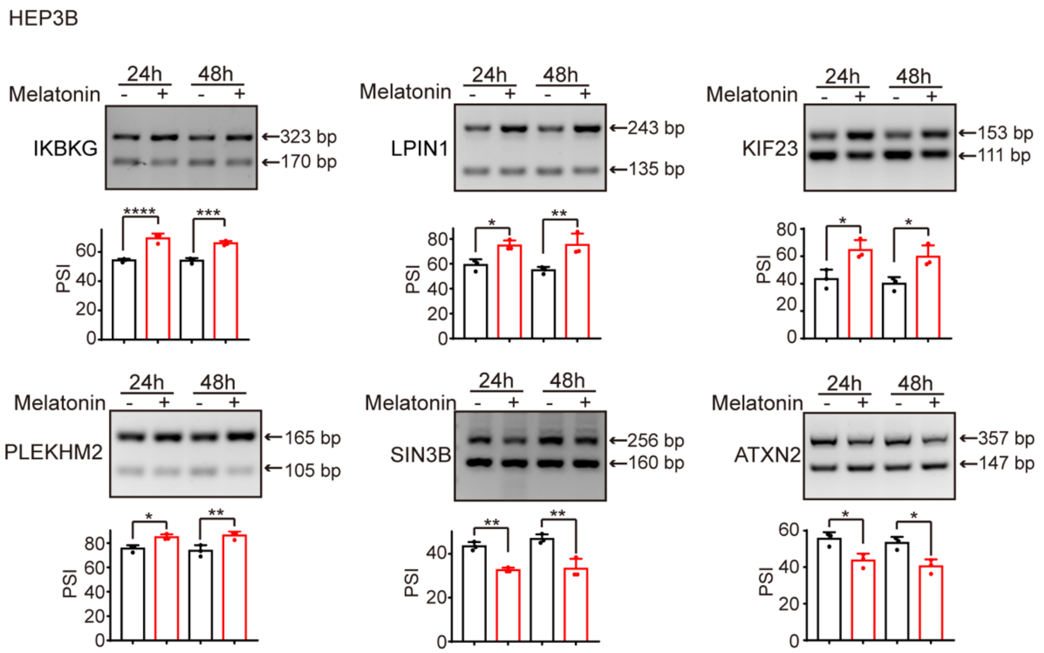

Fig. S4 Alternative splicing switch induced by melatonin in Hep3B cells. Exons skipping in *IKBKG*, *LPIN1*, *KIF23*, *PLEKHM2*, *SIN3B*, and *ATXN2* were examined by semi-quantitative RT-PCR in Hep3B cells treated with 2mM melatonin for 24 h and 48 h.

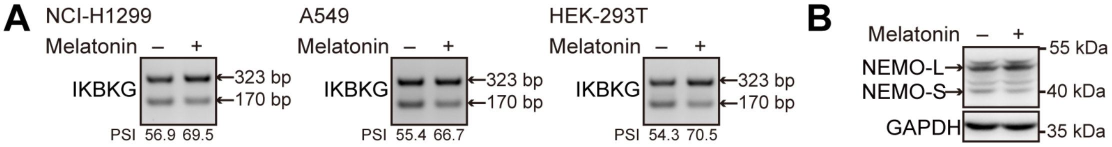

Fig.S5 Melatonin induce alternative splicing change of *IKBKG* in multiple cancer cells. (A) Exons skipping in *IKBKG* were examined by semi-quantitative RT-PCR in NCI-H1299, A549 and HEK293T cells treated with 1mM melatonin. (B) Protein level of endogenous NEMO-L in HepG2 cells treated with melatonin was confirmed by western blot analysis using anti-NEMO antibodies.

Supplementary Table

CRISPLD 2F	AGGATTTGGACTGCTACACG
CRISPLD *2*-R	TTTATCCACGGGCATCACG
SESN3-F	AAAGGGAAGATGAAGAGGCG
SESN3-R	GCTCGGAATGTTGGCAAATG
EFR3B -F	GGAGAATAGGAACCGTCTGAC
EFR3B -R	ATGGTAGAGAACTTGTGGCG
GPCPD1-F	GAGGCAAAAGCTAAGGGACTAG
GPCPD1-R	CAAAGATGAGGGAACAAAGCG
CAPN5-F	CATCTACTGCCACTCCAACTC
CAPN5-R	GCTGGTTCCTCTTAGTCTCATC
PPP1R36 -F	GTACTGTATCCTTGTGCTGGG
PPP1R36 -R	CTCCTGATTCCTCATCTTCACG
CHST9-F	ACCCCAAACAAGTCTTCCTC
CHST9-R	CTCGTACATCCTCAGGCATG
UNC93A-F	TTGTATGGAAAGGTCTCGCAG
UNC93A-R	CTCCCACAGGCGGTAATTG
IKBKG-SE–F	TCTGTGAAAGCCCAGGTGAC
IKBKG-SE–R	CTGCTCTTGATGTGGTTGTCG
LPIN1-SE-F	CCTCAGTCAGCCTCATACCC
LPIN1-SE-R	GTTCTTCTGCCCTGTCCTTTC
ITGA6-SE-F	ATTCGGGAGTACCTTGGTGG
ITGA6-SE-R	CATGGTATCGGGGAACACTG
TERF1-SE-F	GCAGCGGCAAAAGTAGTAGAAAG
TERF1-SE-R	TAAGGTCTTGTTGCTGGGTTCC
KIF23-SE-F	TGATCTATTGGAAGAGGTGC

KIF23-SE-R	ACTTCTGTACATCCTGCAAC
PLEKHM2-SE-F	TGGAGTGGGATGACAGTGCG
PLEKHM2-SE-R	CCTTGCTGCTGGTCAGGTC
SIN3B-SE-F	CGGGAAATTGATTATGCATCC
SIN3B-SE-R	ACGTGGAGTCCTCAGACCA
ATXN2-SE-F	CCATGCCATCAAGATCCACTT
ATXN2-SE-R	AACCCCTCCCAGCAGAAAC
ENTPD6-A3E-F	ATGCGGAGAAGGGAGGCAG
ENTPD6-A3E-R	CTTCAGCACTTTGCTCCTGGG
CLEC16A-A5E-F	AGGATATTCAGAGAAGTTCTGCC
CLEC16A-A5E-R	CGGCGCTTTTCTCCTCGTC
MT1-sh2-F	CCGGGCTGATGTCGATATTTAACAACTCGAGTTGTTAAATATCGACATCAGCTTTTTG
MT1-sh2-R	AATTCAAAAAGCTGATGTCGATATTTAACAACTCGAGTTGTTAAATATCGACATCAGC
MT1-Q-F	CTGCGTCCTCATCTTCACCA
MT1-Q-R	AGATAGCCCAGGTTCCACCC
NEMO-Nhe1-F1	cac GCTAGC AATAGGCACCTCTGGAAGAG
NEMO-Not1-R2	cac GCGGCCGC CTACTCAATGCACTCCATGACAT
